# Risk factors for peripherally inserted central catheter malposition in preterm infants: a multicenter retrospective study

**DOI:** 10.3389/fped.2025.1534799

**Published:** 2025-03-07

**Authors:** Chunyan Liang, Jinming Liu, Yingzi Tang, Wenpiao Zhao, Ruining Xin, Xiaoqun Gu, Fang Huang, Yu'e Lai, Wangjin Huang, Yanhong Liu, Mei Lin, Lili Pan, Guirong Cao, Sudan Tan, Chunliu Wei, Fangyuan Lin

**Affiliations:** ^1^ Nursing Department, Maternal and Child Health Hospital of Guangxi Zhuang Autonomous Region, Nanning, China; ^2^ Neonatal Intensive Care Unit, Maternal and Child Health Hospital of Guangxi Zhuang Autonomous Region, Nanning, China; ^3^ Neonatal Surgery Department, Maternal and Child Health Hospital of Guangxi Zhuang Autonomous Region, Nanning, China; ^4^ Neonatal Intensive Care Unit, Guilin Maternal and Child Health Hospital, Guilin, China; ^5^ Neonatal Intensive Care Unit, Yulin Maternal and Child Health Hospital, Yulin, China; ^6^ Neonatal Intensive Care Unit, Qinzhou Maternal and Child Health Hospital, Qinzhou, China; ^7^ Neonatal Intensive Care Unit, Naning Maternity and Child Health Hospital, Nanning, China; ^8^ Neonatal Intensive Care Unit, Minzu Hospital of Guangxi Zhuang Autonomous Region, Nanning, China; ^9^ Neonatal Intensive Care Unit, The People’s Hospital of HeChi, HeChi, China; ^10^ Neonatal Intensive Care Unit, The First People’s Hospital of Hechi, Hechi, China; ^11^ Neonatal Intensive Care Unit, Bobai County People’s Hospital, Bobai, China

**Keywords:** preterm infants, peripherally inserted central catheter, catheter tip, malposition, risk factors, multicentre study

## Abstract

**Objective:**

To explore the risk factors affecting peripherally inserted central catheter (PICC) tip malposition in preterm infants.

**Methods:**

A retrospective collection of clinical data from preterm infants who underwent PICC placement in the neonatal departments of Guangxi Maternal and Child Health Hospital and eight other hospitals from January 2021 to April 2024 was conducted. The incidence of catheter tip malposition was analyzed. The infants were divided into two groups based on the occurrence of catheter tip malposition: the malposition group and the non-malposition group. Multifactorial logistic regression and multimodel logistic regression analyses were employed to explore the influencing factors of PICC tip malposition in preterm infants.

**Results:**

A total of 1,449 infants were ultimately included in the study, with an incidence of catheter tip displacement of 12.56% (182 out of 1,449). Adjusted results from multimodel regression analysis of covariates indicated that Sample selection location in Guilin (OR = 2.30, 95% CI:1.24∼4.25), Yulin (OR = 4.35, 95% CI: 2.27∼8.34) and Qinzhou (OR = 2.63, 95% CI:1.37∼5.08), duration of catheter insertion procedure (OR = 1.01, 95% CI: 1.01∼1.02), duration of catheter dwell (OR = 1.04, 95% CI: 1.02∼1.07), weight percentile at the time of catheter malpositioning (OR = 11.39, 95% CI:4.81∼26.95), extremely preterm group (<28 weeks gestation) (OR = 4.42, 95% CI: 1.29∼15.16) were risk factors for catheter tip displacement. Additionally, site of PICC catheterization in neck as a risk factor (OR = 3.48, 95% CI: 1.89∼6.40).

**Conclusion:**

Sample selection location in Guilin, Yulin and Qinzhou, duration of catheter insertion procedure, duration of catheter dwell, weight percentile at catheter removal, extremely preterm group (<28 weeks gestation) and site of PICC catheterization in neck may increase the risk of PICC catheter tip malposition.

## 1 Introduction

The Peripherally Inserted Central Catheter (PICC) is a vital “lifeline” for the treatment of extremely low birth weight and very low birth weight preterm infants due to its ability to withstand high osmolarity fluids, minimal vascular irritation, and the potential for long-term indwelling within the body (1, 2).

However, catheter tip malposition is a common complication after PICC placement (3), and the American Intravenous Infusion Nursing Society (INS) proposed the optimal location of the catheter tip in the 2016 edition of the Standards of Practice for Infusion Therapy (Infusion Nursing Society, INS), and for central venous catheters placed through the veins of the upper extremities, the ideal location for the tip of the catheter is at the junction of the superior vena cava and the right atrium. For catheters placed through lower extremity veins, the tip should be located within the inferior vena cava and above the level of the diaphragm to ensure the safety and efficacy of treatment (4).

The malposition of the PICC tip refers to the failure of the catheter tip to be correctly positioned in the aforementioned expected central venous location. Malposition may lead to abnormal catheter function and increase the risk of complications, such as phlebitis, bloodstream infections, thrombosis, and catheter fracture (5, 6). It has been reported that the incidence of PICC malposition in preterm infants ranges from 3.36% to 56.0% (7–10). The occurrence of catheter malposition is associated with a variety of factors, including the infant's limb movement, body weight, and the site of catheter insertion (11–13). Previous research has addressed malposition in patients through positional correction, employing methods such as ultrasound-guided finger pressure and partial head turning (14). However, positional studies in infants are relatively rare. Positional adjustment in infants often encounters poor cooperation, and no reports have been found in such studies. Although current detection technologies, such as ultrasound and intracavitary electrocardiography (3, 15), can reduce the incidence of catheter tip malposition during catheter placement, further research and optimization are still needed in actual operations to more accurately monitor the catheter position in real time and make timely adjustments. The popularity of these technologies is not strong, and multicenter studies on PICC tip malposition in preterm infants are lacking. Therefore, the purpose of this study is to focus on the relatively underdeveloped Guangxi region in Southwest China, to identify and manage the risk factors of tip malposition under existing conditions, so as to reduce the occurrence of PICC malposition.

## 2 Materials and Methods

### 2.1 Study subjects

This study is a multicenter retrospective investigation, selecting preterm infant cases admitted to the neonatology departments of nine hospitals in the Guangxi region from January 2021 to April 2024 as the subjects of study. Inclusion criteria: (1) Gestational age at birth of less than 37 weeks. (2) first-time insertion of a PICC. (3) Completion of the PICC catheterization process from insertion to removal during the hospital stay. (4) Infusion of nutritional fluids for ≥5 days. (5) Infusion of hypertonic fluids (>600 mOsm/L). Exclusion criteria: (1) Duration of catheter placement of less than three days. (2) Discharge, withdrawal of treatment, or death during the period from catheter insertion to removal. (3) Incomplete case information. (4) Unclear chest x-ray images of the catheter tip that prevent identification. This study was approved by the Ethics Committee of the Guangxi Zhuang Autonomous Region Maternal and Child Health Hospital (No.202411-5).

### 2.2 Data collection

(1) General Information: Gender, gestational age at birth, and birth weight. (2) Laboratory Test Indices: Platelet count, high-sensitivity C-reactive protein, and hemoglobin levels prior to catheter insertion. (3) Additional Data: Type of venous catheter used before PICC placement, Sample selection location, days from birth to catheter insertion, corrected gestational age at the time of catheter insertion, weight at the time of catheter insertion, weight percentile at the time of catheter insertion, site of PICC catheterization, duration of catheter insertion procedure, catheter depth at the end of insertion, external catheter length, duration of catheter dwell, corrected gestational age of malpositioning, weight at the time of catheter malpositioning., and weight percentile at the time of catheter malpositioning. (4) Outcome Measure: Malposition of the catheter tip.

### 2.3 Definitions and determination of relevant research variables

(1)Classification of Preterm Infants: Categorized into four groups based on gestational age at birth—Late Preterm Infants (34–<37 weeks), Moderately Preterm Infants (32–<34 weeks), Very Preterm Infants (28–<32 weeks), and Extremely Preterm Infants (<28 weeks) (16). (2) Duration of Catheter Dwell: Defined as the number of days the catheter was retained after detecting mal- positioning. (3) Weight Percentile at Catheter Insertion and Removal: Calculated according to the Fenton growth curves (17). This study is based on Fenton's calculator, where the corresponding values are entered into the module to calculate the percentile of the child's weight (18). (4) Determination of Catheter Tip Positioning and Malposition: Chest x-ray is considered the gold standard for PICC tip positioning (18). According to the 2016 edition of the INS Standards of Practice (4), the ideal tip position when cannulating via an upper limb vein is at the junction of the superior vena cava and the right atrium, typically using the 4th to 6th thoracic vertebrae as the standard localization markers. In cases of cannulation via a lower limb vein, the catheter tip should be located within the inferior vena cava, above the diaphragmatic level, typically using the 8th to 10th thoracic vertebrae as the standard localization markers. Deviation from these positions is defined as malposition. In the present multicenter study, all participating units maintained consistency in the methodology and frequency of monitoring for catheter malpositioning. Specifically, the diagnosis of malpositioning was uniformly conducted via x-ray examination subsequent to catheter placement. The monitoring frequency was established at two time points: the initial x-ray localization was executed within 24 h post-PICC insertion to ensure accurate catheter tip positioning, and the second x-ray localization was documented prior to catheter removal to capture the final position of the catheter.

### 2.4 Data collection methods and quality control

The Guangxi Zhuang Autonomous Region Maternal and Child Health Hospital serves as the coordinating center for the project, overseeing organizational management, quality control, and the integration, entry, and analysis of data. Each participating institution is responsible for collecting medical records from their own hospital. Catheter insertion is performed using the Seldinger technique by two nurses qualified in PICC procedures. The infants are placed in incubators while the nurses perform the puncture at the bedside. In accordance with the neonatal PICC insertion operational standards, a single-lumen PICC catheter with a size of 1.9 F is used for catheter insertion in all cases. To ensure the accuracy, completeness, and professionalism of the data, data collectors underwent unified professional training to standardize and unify the data collection methods. During the data entry and multicenter consolidation stages, each unit adopted a dual-entry method to minimize human errors. Data collection skills: Each unit extracted data from the nurse information system according to the inclusion and exclusion criteria of the study, directly extracting data using the information system. For textual tables, standardized record forms were used to ensure that each piece of data had a clear classification and location. The forms should be clear and easy to read, while also paying attention to the time, unit, and source of the data entry. With the help of Excel tables, each unit's information entry ensured that at least two people entered the data to ensure accuracy. Data management strategy: Data management requires the joint efforts of personnel from different units. The research team has data managers, clinical research assistants, nursing graduate students, etc. After summarizing, cleaning, and analyzing the data, it is distributed again to each unit for verification.

### 2.5 Statistical analysis

Statistical analysis was conducted using R software version 4.3.1. Quantitative data are presented as mean ± standard deviation (SD), and comparisons between groups for quantitative data that conform to a normal distribution were made using the two-sample *t*-test. Quantitative data that do not conform to a normal distribution are expressed as median (M) with interquartile range (Q1, Q3) and were compared using the Mann–Whitney U test. Categorical data are represented by counts and percentages (%), with intergroup comparisons made using the chi-square test. Variables with *P* < 0.2 in univariate analysis were selected for multivariate logistic regression analysis (forward selection method), and multiple model logistic regression analysis was employed for adjustment. The significance level was established at *α* = 0.05.

## 3 Results

### 3.1 General characteristics

**Figure 1:**
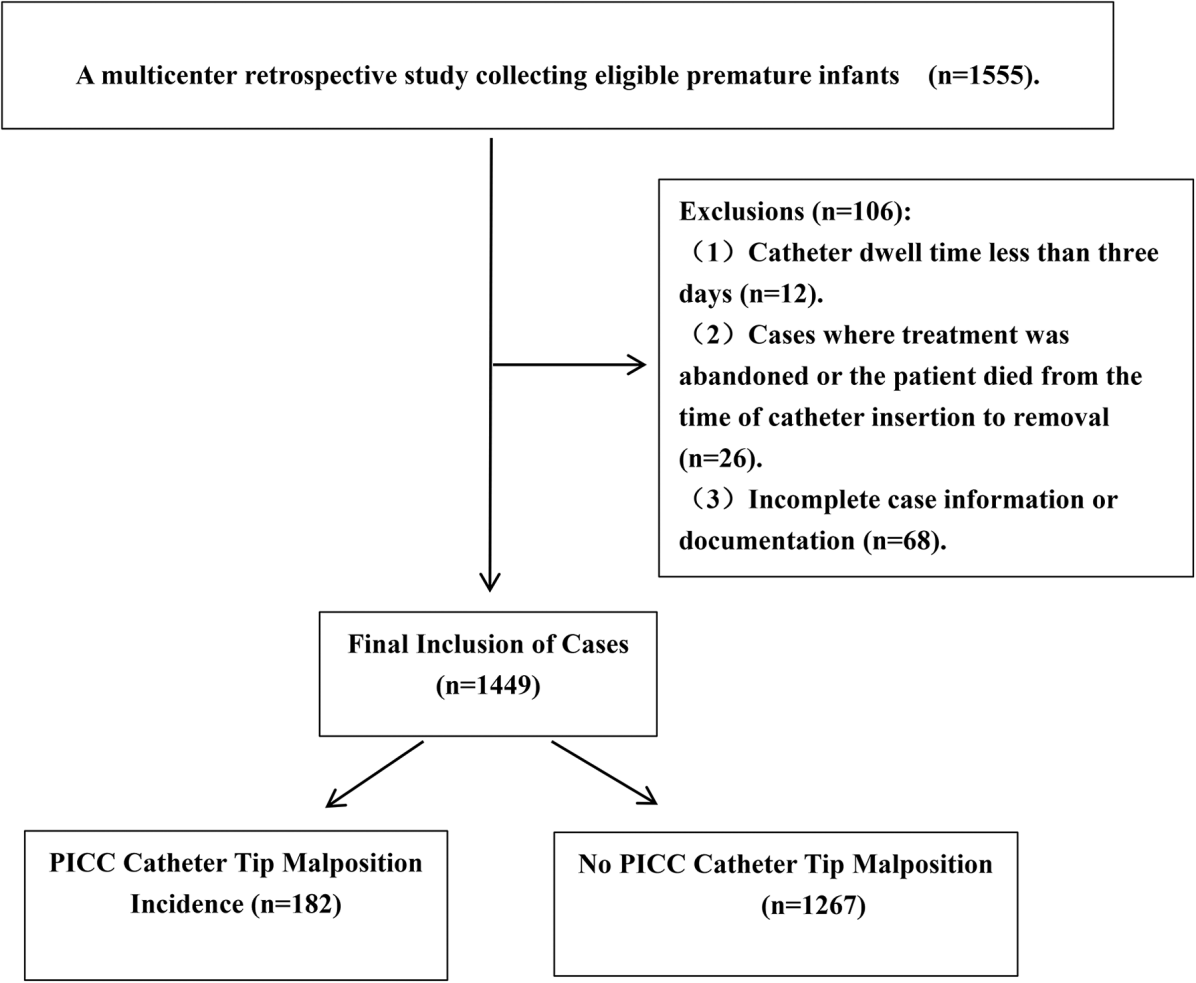
Data cleansing and stratification process of study subjects.

**Figure 2:**
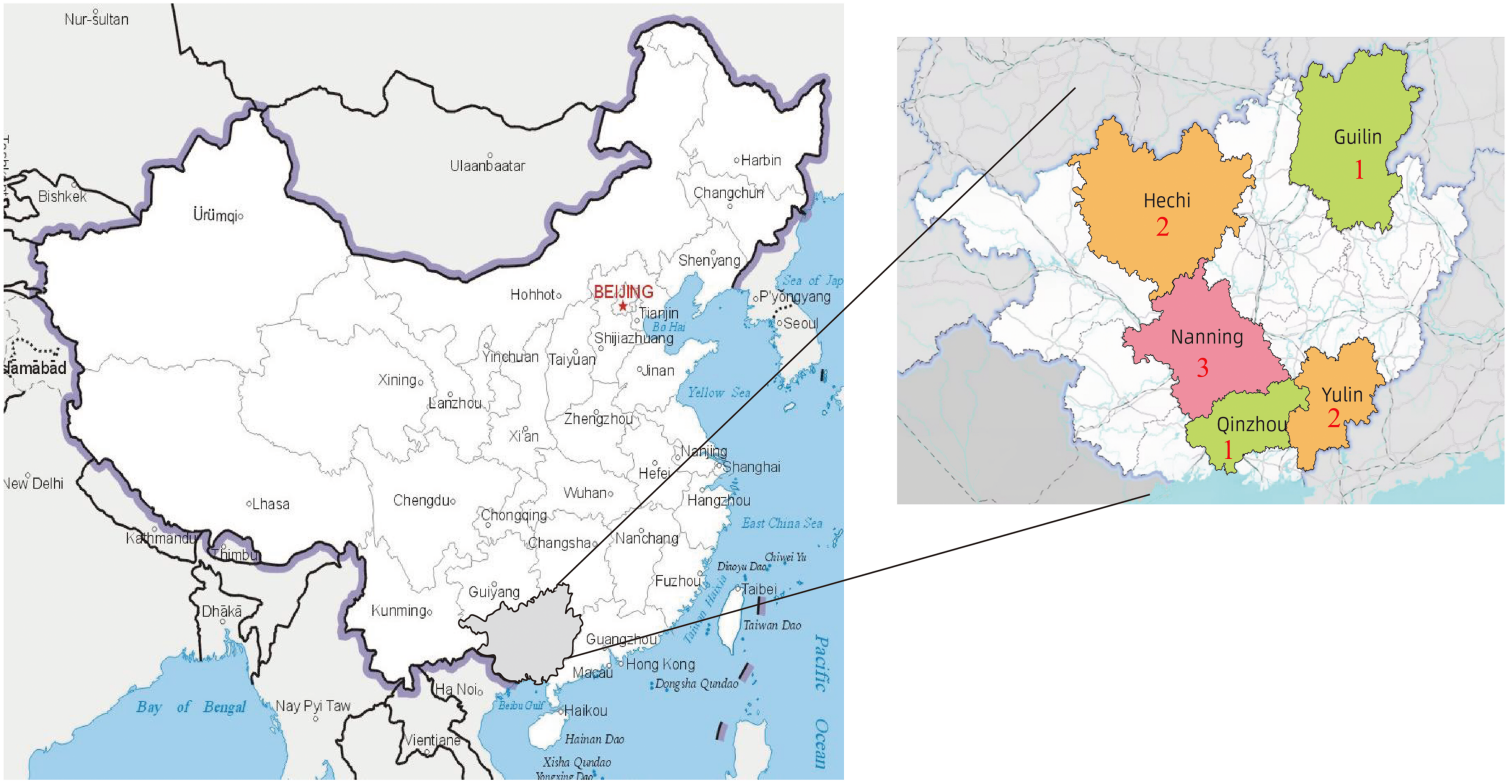
Geographic distribution of the 9 selected units within guangxi region.

In the present study, a preliminary screening was conducted on a total of 1,555 cases with complete data. Based on the inclusion and exclusion criteria, 106 cases were excluded, resulting in a final sample of 1,449 cases included for analysis. Case enrollment across centers: Maternal and Child Health Hospital of Guangxi Zhuang Autonomous Region was 475, and Guilin Maternal And Child Health Hospital with 129 cases, and Yulin Maternal and Child Health Hospital with 218 cases, and the Qinzhou Maternal and Child Health Hospital with 263 cases, and Naning Maternity And Child Health Hospital with 71 cases, and Minzu Hospital of Guangxi Zhuang Autonomous Region with 65 cases, and The First People's Hospital of Hechi, and Hechi with 146 cases, and The People's Hospital of HeChi with 59 cases, and the Bobai County People's Hospital, and Bobai with 23 cases.Among the cases included, there were 828 males (57.14%) and 621 females (42.86%). The mean gestational age was 29.64 ± 2.59 weeks; the average birth weight was (1,211.02 ± 398.31)g; the distribution across preterm categories was as follows: 265 cases (18.29%) in the moderately preterm group (32–<34 weeks), 802 cases (55.35%) in the very preterm group (28–<32 weeks), and 382 cases (26.36%) in the extremely preterm group (<28 weeks); PICC catheter tip malposition occurred in 182 cases (12.56%), while no malposition was observed in 1,267 cases (87.44%). The data cleansing and grouping process of the study subjects is illustrated in (Figure 1), and the nine hospitals were categorized based on their respective cities and used as variables. Specifically, there were three hospitals in Nanning, one in Guilin, two in Yulin, one in Qinzhou, and two in Hechi, with the detailed distribution shown in (Figure 2).

### 3.2 Univariate analysis of PICC malposition

Based on whether PICC malposition occurred, the cases were divided into a malposition group and a non-malposition group. The results of univariate analysis showed that the two groups had statistically significant differences in the Sample selection location, classification of preterm infants, corrected gestational age at the time of catheter insertion, corrected gestational age at the time of catheter removal, weight percentile at the time of catheter insertion, and weight percentile at the time of catheter removal (*P* < 0.05). However, no statistically significant differences were found in Gender, gestational age at birth, and birth weight, as well as Laboratory Test Indices: Platelet count, high-sensitivity C-reactive protein, and hemoglobin levels prior to catheter insertion, and the comparison of related indicators before and after catheter insertion (*P* > 0.05) (Table 1).

**Table 1:** Univariate analysis of the PICC malposition group and the non-malposition group.

Characteristic	No-malposition (*n* = 1,267)	Malposition (*n* = 182)	*χ*^2^/*Z*	*P*
Sample selection location [*n* (%)]			*χ*^2^ = 22.00	**<0**.**001**
Nanning	557 (43.96)	54 (29.67)		
Guilin	107 (8.45)	22 (12.09)		
Yulin	193 (15.23)	48 (26.37)		
Qinzhou	231 (18.23)	32 (17.58)		
Hechi	179 (14.13)	26 (14.29)		
Birth age M (Q₁, Q₃)	29.43 (27.86, 31.43)	29.14 (27.29,30.54)	*Z* = −2.21	**0**.**027**
Site of PICC catheterization [*n* (%)]			*χ*^2^ = 21.10	**<0**.**001**
Upper limbs	588 (46.41)	76 (41.76)		
Lower limbs	504 (39.78)	69 (37.91)		
Head	119 (9.39)	14 (7.69)		
Neck	56 (4.42)	23 (12.64)		
Birth weight M (Q₁, Q₃)	1,150.00 (950.00, 1,385.00)	1,150.00 (920.00, 1,400.00)	*Z* = −0.16	0.871
Gender [*n* (%)]			*χ*^2^ = 0.64	0.423
Male	719 (56.75)	109 (59.89)		
Female	548 (43.25)	73 (40.11)		
Classification of preterm infants [*n* (%)]			*χ*^2^ = 7.90	0.019
Moderately preterm group	241 (19.02)	24 (13.19)		
Very preterm group	706 (55.72)	96 (52.75)		
Extremely preterm group	320 (25.26)	62 (34.07)		
hsCRP [*n* (%)]			*χ*^2^ = 1.70	0.192
≤10 mg/L	105 (8.29)	10 (5.49)		
>10 mg/L	1,162 (91.71)	172 (94.51)		
PLT M (Q₁, Q₃)	209.00 (166.00, 277.00)	210.50 (167.00, 270.75)	*Z* = −0.16	0.872
Hb M (Q₁, Q₃)	136.00 (116.00, 158.00)	138.00 (114.00, 159.75)	*Z* = −0.12	0.907
Days from birth to catheter insertion M (Q₁, Q₃)	6.00 (3.00, 10.00)	7.00 (4.00, 11.00)	*Z* = −0.98	0.329
Corrected age at the time of catheter insertion M (Q₁, Q₃)	30.57 (29.00, 32.43)	30.00 (28.61, 31.43)	*Z* = −2.73	**0**.**006**
Weight at the time of catheter insertion M (Q₁, Q₃)	1,180.00 (960.00, 1,415.00)	1,185.00 (960.00, 1,420.00)	*Z* = −0.33	0.739
Duration of catheter insertion procedure M (Q₁, Q₃)	62.00 (48.50, 78.00)	65.00 (48.00, 80.00)	*Z* = −0.90	0.369
Catheter depth at the end of insertion M (Q₁, Q₃)	14.50 (12.50, 18.50)	15.00 (13.00, 18.00)	*Z* = −0.58	0.564
External catheter length M (Q₁, Q₃)	2.00 (1.00, 2.90)	2.00 (1.00, 2.50)	*Z* = −0.35	0.725
Duration of catheter dwell M (Q₁, Q₃)	20.00 (14.00, 29.00)	20.00 (16.00, 30.75)	*Z* = −2.05	**0**.**040**
Corrected gestational age of malpositioning M (Q₁, Q₃)	32.00 (31.00, 34.00)	31.00 (30.00,33.00)	*Z* = −2.93	**0**.**003**
Weight at the time of catheter malpositioning M (Q₁, Q₃)	1,574.00 (1,292.00, 1,876.00)	1,588.00 (1,292.50, 1,930.50)	*Z* = −0.52	0.606
Weight percentile at the time of catheter insertion M (Q₁, Q₃)	0.14 (0.07, 0.34)	0.21 (0.10, 0.45)	*Z* = −3.52	<0.001
Weight percentile at the time of catheter malpositioning M (Q₁, Q₃)	0.10 (0.03, 0.28)	0.20 (0.06, 0.56)	*Z* = −4.23	**<**.**001**
Type of venous catheter used before PICC placement			*χ*^2^ = 2.20	0.138
Peripheral Intravenous Catheter	776 (61.25)	101 (55.49)		
Umbilical Venous Catheter	491 (38.75)	81 (44.51)		

The bold font indicates *P* < 0.05.

### 3.3 Multivariate logistic regression analysis

In the multivariate logistic regression analysis, variables with *P* < 0.2 from the univariate analysis were included. The variables were coded as follows: Age, corrected gestational age at the time of catheter insertion, duration of catheter dwell, corrected gestational age at the time of catheter removal, weight percentile at the time of catheter insertion, and weight percentile at the time of catheter removal were all treated as continuous variables and entered as their actual values; Sample selection location: Nanning = 1, Guilin = 2,Yulin = 3, Qinzhou = 4, Hechi = 5; preterm status grouping: moderately preterm group = 1, very preterm group = 2, extremely preterm group = 3; site of PICC catheterization: upper limb = 1, lower limbs = 2, head = 3, Neck = 4. The results indicated that sample selection location in Guilin (OR = 2.42, 95% CI: 1.32∼4.42), Yulin (OR = 4.05, 95% CI: 2.21∼7.42) and Qinzhou (OR = 2.79, 95% CI: 1.48∼5.26), Extremely preterm group (OR = 4.17, 95% CI: 1.25∼13.93), site of PICC catheterization in neck (OR = 3.55, 95% CI: 1.95∼6.44), duration of catheter insertion procedure (OR = 1.01, 95% CI: 1.01∼1.02), duration of catheter dwell (OR = 1.04, 95% CI: 1.02∼1.06), Weight percentile at the time of catheter removal (OR = 7.16, 95% CI: 3.47∼14.78) were risk factors for catheter tip malposition (Table 2).

**Table 2:** Logistic regression analysis of factors influencing PICC catheter tip malposition.

Variables	*β*	S.E	*Z*	OR (95% CI)	*P*
Sample selection location					
Nanning				1.00 (Reference)	
Guilin	0.87	0.31	2.82	2.38 (1.30∼4.35)	**0**.**005**
Yulin	1.38	0.31	4.48	4.05 (2.21∼7.42)	**<0**.**001**
Qinzhou	0.99	0.32	3.08	2.79 (1.48∼5.26)	**0**.**002**
Hechi	0.34	0.29	1.17	1.40 (0.80∼2.47)	0.240
Classification of preterm infants					
Moderately preterm group				1.00 (Reference)	
Very preterm group	0.70	0.41	1.70	2.01 (0.90∼4.51)	0.089
Extremely preterm group	1.38	0.62	2.24	3.98 (1.19∼13.30)	**0**.**025**
Type of venous catheter used before PICC placement					0.470
Peripheral intravenous catheter				1.00 (Reference)	
Umbilical venous catheter	−0.14	0.21	−0.68	0.87 (0.58∼1.30)	0.498
hsCRP					0.214
≤10 mg/L				1.00 (Reference)	
>10 mg/L	0.45	0.36	1.24	1.57 (0.77∼3.22)	
Site of PICC catheterization					
Upper limbs				1.00 (Reference)	
Lower limbs	0.33	0.23	1.45	1.39 (0.89∼2.17)	0.148
Head	−0.11	0.33	−0.33	0.90 (0.47∼1.71)	0.741
Neck	1.27	0.30	4.17	3.55 (1.96∼6.44)	**<0**.**001**
Birth age	0.17	0.09	1.85	1.19 (0.99∼1.42)	0.065
Corrected age at the time of catheter insertion	0.01	0.05	0.13	1.01 (0.90∼1.12)	0.900
Weight percentile at the time of catheter insertion	0.13	0.40	0.33	1.14 (0.52∼2.52)	0.739
Duration of catheter insertion procedure	0.01	0.00	1.99	1.01 (1.01∼1.02)	**0**.**046**
Duration of catheter dwell	0.03	0.01	3.32	1.03 (1.01∼1.05)	**<0**.**001**
Corrected gestational age of malpositioning	−0.08	0.06	−1.39	0.92 (0.83∼1.03)	0.164
Weight at the time of catheter malpositioning	1.95	0.37	5.29	7.05 (3.42∼14.54)	**<0**.**001**

OR, odds ratio; CI, confidence interval.

The bold font indicates *P* <60.05.

### 3.4 Multimodel logistic regression analysis

Based on the results of the logistic regression analysis, with catheter dwell time, weight percentile at the time of catheter removal, preterm status, and catheter insertion site as the focal exposure factors, a multimodel regression analysis was employed for covariate adjustment. The results indicated that after adjustment, the final model 3 confirmed the sample selection location in Guilin (OR = 2.30, 95% CI: 1.24∼4.25), Yulin (OR = 4.35, 95% CI: 2.27∼8.34) and Qinzhou (OR = 2.63, 95% CI:1.37∼5.08), duration of catheter insertion procedure (OR = 1.01, 95% CI: 1.01∼1.02), duration of catheter dwell (OR = 1.05, 95% CI: 1.03∼1.07), weight percentile at catheter removal (OR = 12.28, 95% CI: 5.16∼29.21), very preterm group (28–<32 weeks gestation) (OR = 2.40, 95% CI: 1.04∼5.54), and extremely preterm group (<28 weeks gestation) (OR = 4.68, 95% CI: 1.36∼16.06) were risk factors for catheter tip displacement. Additionally, Site of PICC catheterization in neck as a risk factor (OR = 3.48, 95% CI: 1.89∼6.40).as risk factors for catheter tip malposition (Table 3).

**Table 3:** Multimodel logistic regression analysis of factors affecting PICC catheter tip malposition.

Variables	Model1		Model2		Model3	
	OR (95% CI)	*P*	OR (95% CI)	*P*	OR (95% CI)	*P*
Sample selection location						
Nanning	1.00 (Reference)		1.00 (Reference)		1.00 (Reference)	
Guilin	2.12 (1.24∼3.63)	**0**.**006**	2.34 (1.28∼4.27)	**0**.**006**	2.26 (1.22∼4.18)	**0**.**009**
Yulin	2.57 (1.68∼3.91)	**<0**.**001**	3.90 (2.25∼6.76)	**<**.**001**	4.18 (2.19∼7.99)	**<**.**001**
Qinzhou	1.43 (0.90∼2.27)	0.131	2.47 (1.32∼4.62)	**0**.**005**	2.55 (1.32∼4.90)	**0**.**005**
Hechi	1.50 (0.91∼2.46)	0.111	1.64 (0.92∼2.91)	0.092	1.59 (0.84∼2.99)	0.151
Classification of preterm infants						
Moderately preterm group	1.00 (Reference)		1.00 (Reference)		1.00 (Reference)	
Very preterm group	1.37 (0.85∼2.19)	0.194	2.23 (0.98∼5.09)	0.056	2.30 (1.00∼5.32)	0.050
Extremely preterm group	1.95 (1.18∼3.21)	**0**.**009**	4.08 (1.21∼13.82)	**0**.**024**	4.42 (1.29∼15.16)	**0**.**018**
Duration of catheter insertion procedure	1.00 (1.00∼1.01)	0.195	1.01 (1.01∼1.02)	**0**.**048**	1.01 (1.01∼1.02)	**0**.**034**
Weight percentile at the time of catheter malpositioning	6.43 (3.70∼11.17)	**<0**.**001**	9.42 (4.10∼21.63)	**<0**.**001**	11.39 (4.81∼26.95)	**<0**.**001**
Duration of catheter dwell	1.01 (1.00∼1.02)	0.151	1.04 (1.02∼1.07)	**<0**.**001**	1.04 (1.02∼1.07)	**<0**.**001**
Site of picc catheterization						
Upper limbs	1.00 (Reference)		1.00 (Reference)		1.00 (Reference)	
Lower limbs	1.06 (0.75∼1.50)	0.745	1.38 (0.88∼2.16)	0.155	1.40 (0.79∼2.47)	0.253
Head	0.91 (0.50∼1.66)	0.760	0.88 (0.46∼1.67)	0.693	0.87 (0.45∼1.69)	0.690
Neck	3.18 (1.85∼5.46)	**<0**.**001**	3.41 (1.87∼6.21)	**<0**.**001**	3.48 (1.89∼6.40)	**<0**.**001**

Model1: Crude.

Model2: adjusted birth age, gender, birth weight, corrected age at the time of catheter insertion, weight at the time of catheter insertion, corrected gestational age of malpositioning, weight at the time of catheter malpositioning.

Model3: adjusted birth age, gender, birth weight, corrected age at the time of catheter insertion, weight at the time of catheter insertion, days from birth to catheter insertion, corrected gestational age of malpositioning, weight at the time of catheter malpositioning, duration of catheter dwell, catheter depth at the end of insertion, external catheter length, type of venous catheter used before PICC placement, hsCRP, PLT, Hb.

OR, odds ratio; CI, confidence interval.

The bold font indicates *P* < 0.05.

## 4 Discussion

The role of PICC in providing venous nutrition and treating diseases in preterm infants is significant (19, 20). However, the growth and development of the infants, along with their activities in daily life, may subject the catheter to external forces. Particularly, the movement of the limb in which the catheter is placed can increase the risk of catheter displacement (12). Research has found that different catheter insertion sites and the selection of veins, due to their distinct anatomical locations, can have varying impacts on catheter malposition. The frequent activity of the upper limbs and different postures, such as abduction and adduction, may also potentially increase the likelihood of catheter malposition (21, 22). If the catheter tip is improperly positioned, being too deep may lead to serious complications such as arrhythmias, pleural effusion, and increased pericardial pressure (23), while being too shallow may result in malposition into other veins.

In our study, the incidence of catheter malposition was 12.56%, which is consistent with the range of 3.6%–56.0% reported in foreign studies. After adjustment for covariates, the results of the multivariate analysis revealed that Sample selection location in Guilin, Yulin and Qinzhou, duration of catheter insertion procedure, the duration of catheter dwell, weight percentile at the time of catheter removal, membership in the very preterm group, and the extremely preterm group, as well as site of PICC catheterization in neck, may be primary influencing factors for catheter malposition. In the study results, the 9 units were from four cities in Guangxi, with Guilin, Yulin, and Qinzhou showing higher rates of malposition compared to Nanning. Nanning, as the capital of Guangxi, is home to three hospitals, two of which are pediatric specialty hospitals. The medical conditions and nursing standards in these hospitals are relatively high. Therefore, it is likely that the lower malposition rate is influenced by political and geographical factors. However Long-term in dwelling catheters increase unfavorable factors for catheter safety, and catheter malposition may be associated with the duration of catheter dwell. Studies have found that malposition can occur as early as three days after catheter insertion (24), with a malposition rate as high as 83% (25). In this study, the median indwelling duration of the catheter in the malposition group reached 20 days, which is approximately three weeks. This aligns with the findings of Tao (15), who reported that catheter malposition occurs approximately 3 weeks after insertion. Therefore, greater caution should be exercised with catheters that are indwelt for longer periods, highlighting the necessity of early tracking of catheter tip position post-insertion. Currently, chest x-rays are widely used for localization in clinical settings, but repeated exposure to x-rays can cause unnecessary harm. Although alternative x-ray localization methods exist, such as the use of ultrasound (26) and intracavitary electrocardiography (EC-ECG) (3, 27), which reduce x-ray exposure to some extent, they require certain imaging knowledge for support. EC-ECG is susceptible to external interference and has lower accuracy compared to standard ultrasound, and its adoption is currently limited. The Neonatal PICC Operation and Management Guidelines (2021) (28) indicate that x-ray and ultrasound technologies are strongly recommended for catheter tip localization, while EC-ECG is weakly recommended. Combining the advantages of these methods, future research should focus on monitoring catheter malposition using x-ray in conjunction with ultrasound to meet the dynamic observation needs of long-term catheterization in preterm infants and to achieve effective early intervention in identifying the risk of PICC catheter tip displacement. Therefore, clinical practice should enhance the training of neonatologists in bedside ultrasound techniques and promote multidisciplinary collaboration to expand the application of this technology in observing PICC tip positions, enabling timely detection and management of catheter malposition and reducing complications arising from catheter displacement.

This research found that the extremely preterm group (OR = 4.42, 95% CI: 1.29∼15.16) have a higher risk of catheter malposition; univariate analysis results showed that the weight percentile at the time of catheter insertion and removal were both statistically significant. After adjustment for variables in the multivariate regression model, it was found that the weight percentile at the time of catheter removal is an independent risk factor for catheter malposition. It was found that in the third to fifth week after PICC catheter placement, the catheter position moved 1.5–3.0 centimeters with weight gain (15).

Zhang M et al. (29) also confirmed that with the increase in growth and development of very low birth weight infants, the risk of catheter malposition increases. For the group of small for gestational age (SGA) infants, the weight gain is associated with PICC tip displacement (*P* < 0.05), with a 10%, 35%, and 55% increase in weight of SGA infants being key points for detecting catheter malposition, warranting re-localization with x-ray. Therefore, in the management of PICC catheters, catheter monitoring should be intensified for preterm infants with a gestational age of less than 32 weeks to detect secondary catheter tip malposition at an early stage.

The present research, among the same group of catheter placements, the highest rate of malposition occurred in other sites. The results of the multivariate logistic regression showed that catheterization in other venous sites (excluding upper and lower limb veins) was associated with an increased risk of catheter tip malposition (OR = 1.89, 95% CI: 1.20∼2.98), with an 89% increase in risk compared to upper limb catheterization. This may be related to the special catheterization sites in other parts of the body, such as the neck and head. For instance, the internal jugular vein is more challenging to cannulate due to the large head and short neck of infants (30), and securing the catheter post-insertion is also more difficult. Additionally, routine maintenance of the catheter is less convenient, and frequent dressing changes may be required due to sweating. Therefore, when the PICC catheterization site in preterm infants is not in the upper or lower limb veins, closer monitoring of the catheter tip position is warranted. Furthermore, since the malposition rate in this study includes both primary malposition (malposition occurring within 24 h) and secondary malposition (malposition occurring after 24 h post-catheterization), the results may also be related to spontaneous correction after catheter malposition. Studies have shown that when upper limb catheterization is chosen, and the catheter tip is malpositioned to the internal jugular vein, external jugular vein, and submandibular vein, the spontaneous repositioning rates within 24 h can reach 71.4%, 60.0%, and 50.0%, respectively (31, 32). This suggests that spontaneous correction may occur after catheter malposition, and x-ray imaging before catheter removal, due to the longer time span, may not capture the malposition. Future prospective studies could further validate this finding from our study.In the event of catheter malposition, when positional adjustments are ineffective, the catheter may temporarily continue to function as a peripheral venous catheter within a short-term period (33). For short-term therapeutic interventions, even if the catheter is partially malpositioned, such as being located in the internal jugular vein or subclavian vein, it can still be utilized after a thorough risk-benefit assessment (34). However, close monitoring is imperative to prevent the occurrence of complications such as phlebitis and infection.

### 4.1 Limitations

It should be noted, however, that the findings of this study are not without certain limitations. The design as a retrospective multicenter study may be susceptible to biases and confounding effects. For example, the limited observation time points for catheter malposition may not capture all instances of displacement, which could vary in occurrence. Future research intends to employ prospective multicenter studies that utilize ultrasound localization to monitor malposition throughout the entire period of catheterization. This approach will be tailored to neonates with varying gestational ages and weight gain trajectories, with the aim of ensuring the safety of PICC management in preterm infants and mitigating the risk of catheter malposition.

## 5 Conclusions

This study employed a multicenter retrospective approach to analyze the incidence and determinants of PICC tip malposition.The findings revealed that Sample selection location in Guilin, Yulin and Qinzhou, duration of catheter insertion procedure, duration of catheter dwell, weight percentile at the time of catheter malpositioning, extremely preterm group (<28 weeks gestation) and site of PICC catheterization in neck, are pivotal factors influencing PICC catheter tip displacement. Specifically, an extended duration of catheter indwelling is associated with an increased risk of catheter tip malposition. A rise in weight percentile correlates significantly with a heightened risk of catheter tip displacement, extremely preterm infants, particularly those born at less than 28 weeks of gestation, face a greater risk of catheter malposition, and the selection of catheterization sites other than the upper and lower limb veins is linked to an elevated risk of catheter tip malposition.

## Data Availability

The data analyzed in this study is subject to the following licenses/restrictions: the dataset used or analyzed during the current study is available from the corresponding author upon reasonable request. Requests to access these datasets should be directed to Fang Huang, 2044932385@qq.com or Yu'e Lai, 542773627@qq.com.
